# Personal, social, and environmental correlates of physical activity in adults living in rural south-west England: a cross-sectional analysis

**DOI:** 10.1186/1479-5868-10-129

**Published:** 2013-11-21

**Authors:** Emma Solomon, Tim Rees, Obioha C Ukoumunne, Brad Metcalf, Melvyn Hillsdon

**Affiliations:** 1 Sport and Health Sciences, College of Life and Environmental Sciences, University of Exeter, St. Luke’s Campus, Heavitree Road, Exeter EX1 2LU, UK; 2 PenCLAHRC, University of Exeter Medical School, Veysey Building, Salmon Pool Lane, Exeter EX2 4SG, UK

**Keywords:** Physical activity, Rural communities, Cross-sectional study, Multilevel modelling

## Abstract

**Background:**

Despite the health risks, physical inactivity is common. Identifying the correlates of physical activity to inform the design of interventions to reduce the disease burden associated with physical inactivity is a public health imperative. Rural adults have a unique set of characteristics influencing their activity behaviour, and are typically understudied, especially in England. The aim of this study was to identify the personal, social, and environmental correlates of physical activity in adults living in rural villages.

**Methods:**

The study used baseline data from 2415 adults (response rate: 37.7%) participating in the first time period of a stepped-wedge cluster randomised trial, conducted in 128 rural villages from south-west England. Data collected included demographic characteristics, social factors, perception of the local environment, village level factors (percentage male, mean age, population density, Index of Multiple Deprivation, and sport market segmentation), and physical activity behaviour. Random effects (“multilevel”) logistic regression models were fitted to the binary outcome whether individuals met physical activity guidelines, and random effects linear regression models were fitted to the continuous outcome MET-minutes per week leisure time physical activity, using the personal, social, environmental, and village-level factors as predictors.

**Results:**

The following factors both increased the odds of meeting the recommended activity guidelines and were associated with more leisure-time physical activity: being male (p = 0.002), in good health (p < 0.001), greater commitment to being more active (p = 0.002), favourable activity social norms (p = 0.004), greater physical activity habit (p < 0.001), and recent use of recreational facilities (p = 0.01). In addition, there was evidence (p < 0.05) that younger age, lower body mass index, having a physical occupation, dog ownership, inconvenience of public transport, and using recreational facilities outside the local village were associated with greater reported leisure-time physical activity. None of the village-level factors were associated with physical activity.

**Conclusions:**

This study adds to the current literature on the correlates of physical activity behaviour by focusing on a population exposed to unique environmental conditions. It highlights potentially important correlates of physical activity that could be the focus of interventions targeting rural populations, and demonstrates the need to examine rural adults separately from their urban counterparts.

## Background

Physical inactivity is ranked the fourth leading risk factor for global mortality, to which six percent of all deaths are attributable [1]. Strong evidence supports the direct relationship between physical inactivity and all-cause mortality, coronary heart disease, high blood pressure, stroke, diabetes, obesity, metabolic syndrome, colon cancer, breast cancer, and depression [2]. In fact, to reduce the risk of these diseases, adults are recommended to undertake a minimum of 150 minutes of at least moderate-intensity activity per week [2,3]. Despite this, in England, only 29% of women and 39% of men reported doing sufficient physical activity [4], and physical inactivity is costing the United Kingdom National Health Service in excess of £0.9 billion per year [5]. Additionally, in Devon, south-west England, 17% of all deaths in 2010 could have been prevented if all adults were physically active [6].

Therefore, understanding the factors that explain why some adults are regularly active while others are inactive is of utmost importance to public health research in the United Kingdom [3]. Physical activity is a complex behaviour determined by the interaction of a large number of personal, social, and environmental factors specific to populations, setting, and type of physical activity [7,8]. Furthering the understanding of the factors that influence physical activity behaviour in specific populations will aid the development of effective, tailored intervention strategies aimed at increasing the population prevalence of physical activity.

The majority of physical activity studies to date have examined urban populations [9]. When examining the influence of residential location on physical activity, most studies have found that rural adults are less likely to meet recommended physical activity guidelines than urban adults, making rural residents appropriate targets for future physical activity interventions [10-14]. Several studies have highlighted differences between urban and rural adults. For instance, Parks, Housemann, and Brownson [12] found noticeable differences in the importance of places to exercise on physical activity behaviour. Access to parks, walking trails, and exercise equipment was found to be important for urban adults, while access to neighbourhood streets for activity, and an indoor gym were more important for rural adults [12]. Younger age, fewer barriers to leisure time activity, and social support have been reported as correlates of physical activity in urban women, compared to higher educational attainment and the presence of enjoyable scenery for rural women [11]. Residents of rural areas are also more likely than their urban/suburban counterparts to report lower social support, limited access to exercise facilities, and fewer pavements as barriers to being physically active [11,12]. Eyler [15] found that the most frequently reported barrier to being physically active among rural women was the remoteness and how rural the local area was although neither of these factors were associated with reported activity. Previous research has indicated that being too far from activity facilities is a major barrier for women living in rural areas [16,17]. Most studies that have focused on rural areas have examined communities from the United States, where it is often the case that rural dwellers are of lower socioeconomic status than urban residents [18], which may explain some of the differences in physical activity behaviour compared to urban areas. Generally in England, however, people living in rural areas are often among the most affluent [19]. Across the south-west of England, out of the 300 most deprived areas only 11 were classified as rural [20]. Regardless, it is clear that rural populations face a unique set of challenges associated with physical activity behaviour, and they are clearly understudied in the United Kingdom. Little is known about the correlates of physical activity in adults living in rural villages in the United Kingdom and whether they are different from the correlates reported by urban residents.

The aim of this study was to identify the correlates of physical activity behaviour in adults residing in rural villages in south-west England. The association of personal, social, perceived environmental, and village level factors with self-reported physical activity outcomes was examined.

## Methods

### Recruitment and participants

This study uses baseline data from the first time period of a stepped wedge cluster randomised controlled trial evaluating the effectiveness of a community-level physical activity intervention [21]. The study was conducted in 128 rural villages across Devon, south-west England, each with a population size between 500 and 2000 people. These criteria were set so that villages were large enough to have local facilities suitable for physical activity, but limited in the amount of activity opportunities they offered. The addresses of all households in Devon were purchased from a private company (Address List Utility, Arc en Ciel, Version 3.1 PAF Quarter 1, 2011) and used to generate a complete list of all households within the study villages. From the list, a random sample of households, stratified by village, was selected to receive a survey questionnaire via the post. Households were sent a questionnaire, a participant information sheet and a prepaid return envelope. The adult in each household who had most recently had a birthday was invited to complete the survey. Eligible participants were aged 18 years or over and resident in the household.

The survey consisted of 28 questions and took participants approximately 10–15 minutes to complete, based on estimates obtained during pilot work. Informed consent was implied when participants returned a completed questionnaire. In total, 2,415 adults aged 18 to 102 years returned a questionnaire and formed the sample for the study.

### Measures

#### Physical activity

Physical activity was measured using the self-administered, short version of the International Physical Activity Questionnaire (IPAQ-SV) [22]. The IPAQ-SV includes seven items collecting information on the frequency and duration of physical activities undertaken in the previous seven days (vigorous-intensity activity, moderate-intensity activity, walking, and sitting behaviour). The IPAQ-SV has been rigorously tested for reliability and validity [22-24].

Participants were categorised according to whether they did sufficient physical activity to meet the current United Kingdom physical activity guidelines (at least 150 minutes of moderate-intensity activity per week in bouts of 10 minutes or more, or at least 75 minutes of vigorous-intensity activity per week: [3]). Physical activity level was also analysed using metabolic equivalent (MET) values to calculate participants’ total MET-minutes per week of moderate intensity walking, moderate intensity physical activity, and vigorous intensity physical activity, using the IPAQ-SV scoring methods for calculating physical activity levels [25].

#### Demographic characteristics

Participants were asked to report their gender, age, health status, dog ownership, number of children, and cars in the household. These were based on questions from the Health Survey for England [4], apart from the dog ownership question that was taken from an Australian cohort study [26]. Body mass index (BMI), defined as weight (kg) divided by height squared (m^2^), was calculated from participants’ self-reported height and weight.

#### Social factors

To assess social factors, measures were created based on a multi-national motivation for change scale [27], and a scale developed for use in an Australian cohort study [26] (Table 1). For the ‘commitment to doing more physical activity’ variable, the mean was calculated across the three constituent items, and the resulting variable was categorised based on the tertiles (low, moderate, and high). The mean scores were calculated from the constituent items for the ‘physical activity social norms’, ‘physical activity habit’, and ‘physical activity village supportiveness’ variables, and then categorised into “Unfavourable” (<0), “Neutral” (0), and “Favourable” (>0).

**Table 1:** Survey measures

**Psychosocial factors**	
Commitment to doing more physical activity (3 items – rated from 0 “not at all” to 10 “very much so” [28])	
	How important is it for you to do more physical activity than you do now?
	How confident are you that you could do more physical activity if you decided to?
	To what extent are you trying to do more physical activity?
Physical activity social norms (2 items – rated from -2 “strongly disagree” to +2 “strongly agree” [25])	
	My family is interested in physical activity/sport
	People around my village all seem to be exercising these days
Physical activity habit (3 items - rated from -2 “strongly disagree” to +2 “strongly agree” [25])	
	I find it easy to have a go at physical activities
	I have always done some kind of physical activity
	In the last 2 years, I have been involved in regular physical activity at one time or another
Physical activity village supportiveness (3 items - rated from -2 “strongly disagree” to +2 “strongly agree” [25])	
	I have recently had opportunities to get involved in physical activity
	My village is a good place to be physically active
	There are very few opportunities to be physically active in my village
**Perceived local environmental characteristics**	
***Perceptions of the local area (5 factors)***	
	Traffic and pleasantness of surroundings (4 items - rated from -2 “strongly disagree” to +2 “strongly agree” [26])
	It is pleasant to walk in the local area
	There is a lot of traffic noise in the local area
	There is little traffic in the local area
	It is safe to cross the road in the local area
	Proximity and convenience of walking (4 items - rated from -2 “strongly disagree” to +2 “strongly agree” [26])
	There is a park within walking distance
	The nearest shops are too far to walk to
	There are no convenient routes for walking in the local area
	There are no pavements in the local area
	Safety and convenience of cycling (2 items - rated from -2 “strongly disagree” to +2 “strongly agree” [26])
	The roads are dangerous for cyclists in the local area
	There are convenient routes for cycling in the local area
	Convenience of public transport (1 item - rated from -2 “strongly disagree” to +2 “strongly agree” [26])
	Safety of walking after dark (1 item – rated from -2 “strongly disagree” to +2 “strongly agree” [26])
***Presence of recreational facilities within the local area (3 factors)***	
	Manmade sports facilities in local area (4 items – responses 1 “yes” versus 2 “no” [25])
	Sporting club/recreation centre/gym
	Public swimming pool
	Public tennis/squash courts
	Indoor sports facilities (e.g., sports hall)
	Natural activity facilities in local area (3 items – responses 1 “yes” versus 2 “no” [25])
	Walking routes/footpaths
	Local park/public green space
	River/beach/waterfront
	Community centre/village hall in local area (1 item – responses 1 “yes” versus 2 “no” [25])
Use of recreational facilities (8 items – responses 0 “no, not in the last year”, 1 “yes, in last 12 months” or 2 “yes, in last month” [27])	
	Walking routes/footpaths
	Local park/public green space
	Sporting club/recreation centre/gym
	River/beach/waterfront
	Public swimming pool
	Public tennis/squash courts
	Indoor sports facility (e.g., sports hall)
	Community centre/village hall
Locality of facilities used (8 items – response box for participant to name location of facility used [27])	
	Walking routes/footpaths
	Local park/public green space
	Sporting club/recreation centre/gym
	River/beach/waterfront
	Public swimming pool
	Public tennis/squash courts
	Indoor sports facility (e.g., sports hall)
	Community centre/village hall

#### Perceived local environmental characteristics

Perceived local environmental characteristics were measured using items previously developed for use in a United Kingdom health study, and found to have acceptable levels of test-retest reliability [28] (Table 1). Perceived proximity and use of different recreational facilities were measured in the survey using scales that were previously found to have acceptable test-retest reliability [26,29] (Table 1). The means were calculated from the constituent items for the variables measuring ‘traffic and pleasantness of surroundings’, ‘proximity and convenience of walking’, ‘safety and convenience of cycling’, ‘convenience of public transport’, and ‘safety of walking after dark’, and were then categorised into “Unfavourable” (<0), “Neutral” (0), and “Favourable” (>0).

#### Village-level factors

Five village-level factors were examined: population density [30], mean age of villagers [31], percent of villagers that were male [30], Indices of Multiple Deprivation (higher scores indicates more deprived [32]), and the dominant Sport England Market Segmentation for each village [33]. The Sport England Market Segmentation divides the English adult population into 19 market segments based on their sports participation, motivations, and barriers to doing more sport, allowing Local Authorities, Sport National Governing Bodies and sports clubs to profile both individuals and areas.

### Sample size

Power calculations were based on the intervention study [21]. It was estimated that 10 participants would need to be recruited from each of the 128 villages at each stage of the stepped wedge trial, in order to achieve 80% power at the 5% significance level, based on detecting an increase from 25% to 30% in the proportion of participants that met the recommended activity guidelines [34]. A recent pilot for a population study of travel behaviour in the United Kingdom achieved a response rate of 17% for a short questionnaire postal survey [35]. Using this as a guide, 50 surveys were sent out to each of the 128 villages, anticipating that we would obtain at least 10 responses per village. If the number of completed questionnaires returned within three weeks of the initial mailing was insufficient for a given village, additional questionnaires were sent out to new households.

### Statistical analysis

Random effects (“multilevel”) logistic regression was used to examine whether the personal, social, environmental, and village-level factors were associated with meeting the recommended physical activity guideline (binary outcome). Random effects linear regression was used to study the relationship of the same factors with MET-minutes of moderate-vigorous physical activity per week (continuous outcome). These methods take account of correlation between responses of participants in the same village (clustering). Firstly, crude (unadjusted) models were fitted separately for each factor as the sole predictor in the analysis. Partially adjusted models were then fitted for each type of factor, using as predictors those that were significant at the 5% level in the unadjusted analyses (e.g., a model was fitted with significant personal factors only). Finally, a single fully adjusted model was fitted including all factors of all types that were significant predictors in the partially adjusted models. Only estimates from the unadjusted and fully adjusted models are reported. The tabulated findings are based on analyses of males and females together. Tests of interaction were carried out to assess evidence of differential effects between the gender groups and where found these are commented on in the text. All analyses were carried out using Stata 12.1 software [36].

## Results

Initially, 6,400 surveys were sent out, with an additional 10 surveys sent out after three weeks because two villages had not achieved their quota of 10 completed responses. The median number of completed responses per village was 18 (range 11 to 31). 2415 responses were received in total, achieving a response rate of 37.7%. The majority of respondents were female (62.7%), with a mean (SD) age of 58 years (15.2). Compared to the general population of the study villages, the study participants tended to be older (70.2% versus 59.2% aged 50 years or over), and a greater proportion were female (62.7% versus 51%). The study participants were equivalent to the general village population in terms of their Index of Multiple Deprivation scores (mean (SD) 15.8 (4.0) for both study sample and general village population). The study participants were also extremely similar to the general population in terms of the population density of the village they resided within (mean (SD) 0.62 (0.5) for the study population versus 0.64 (0.6) for the village population). Half of the participants (49.4%) were classified as either overweight or obese, and 66.9% of all respondents reported doing sufficient physical activity to meet the recommended guidelines, reporting a median (interquartile range) total MET-minutes of physical activity per week of 1,638 (0 to 3879; Table 2).

**Table 2:** Descriptive characteristics (N = 2415)

**Personal factors**	**%**
Males	37.3
Age, y	
*18-34*	6.8
*35-49*	23.5
*50-64*	35.7
*65+*	34.5
BMI, kg/m2	
*Normal weight (18–25)*	50.6
*Overweight (25–29.99)*	35.6
*Obese (≥30)*	13.8
Health	
*Poor/fair*	17.9
*Good*	33.9
*Very good/excellent*	48.2
Participants with a long-term illness or disability	28.7
Full-time education leaving age, y	
*16 & under*	37.6
*17-18*	25.8
*19+*	36.6
Occupational activity	
*Not employed*	49.8
*Sedentary/standing job*	36.1
*Physical job*	14.2
Cars in household	
*No cars*	3.9
*1 car*	38.5
*2 or more cars*	57.5
Households with children U15	21.5
Households with dogs	39.2
**Social factors**	
Commitment to doing more physical activity (tertiles)	
*Low*	35.9
*Moderate*	34.1
*High*	30.1
Physical activity social norms	
*Unfavourable*	27.5
*Neutral*	23.4
*Favourable*	49.1
Physical activity habit	
*Unfavourable*	23.8
*Neutral*	8.5
*Favourable*	67.7
Physical activity village supportiveness	
*Unfavourable*	49.2
*Neutral*	22.8
*Favourable*	28.1
**Environmental factors**	
Traffic and pleasantness of surroundings	
*Unfavourable*	12.6
*Neutral*	12.3
*Favourable*	75.2
Proximity and convenience of walking	
*Unfavourable*	37.2
*Neutral*	15.7
*Favourable*	47.1
Safety and convenience of cycling	
*Unfavourable*	32.8
*Neutral*	36.9
*Favourable*	30.3
Convenience of public transport	
*Unfavourable*	59.0
*Neutral*	13.9
*Favourable*	27.2
Safety of walking after dark	
*Unfavourable*	26.2
*Neutral*	16.0
*Favourable*	57.8
Manmade sports facilities in local area (at least one)	61.3
Natural activity facilities in the local area (at least one)	97.3
Community centre/village hall in local area	79.1
Use of recreational facilities	
*No facilities used*	6.5
*Used in last year only*	9.5
*Used in last month*	84.0
Locality of facilities used	
*No facilities used*	15.2
*Local village only*	24.2
*Outside local village only*	13.7
*Both local and not local*	46.9
**Village-level factors**	
Population density (residents per hectare), mean (SD)	0.6 (0.5)
Mean age, (SD)	45.8 (3.3)
% males in village (tertiles)	
*Low*	35.6
*Moderate*	32.1
*High*	32.4
Indices of Multiple Deprivation (IMD)	
*More deprived than median score for villages in Devon*	49.6
Sport England Segmentation	
*3 (Chloe)*	6.9
*6 (Tim)*	48.8
*8 (Jackie)*	0.5
*11 (Philip)*	2.0
*13 (Roger & Joy)*	10.3
*17 (Ralph & Phyllis)*	27.2
*19 (Elsie & Arnold)*	4.3
**Physical activity**	
Meets recommended guidelines	66.9
MET-minutes/week (total LTPA), median (IQR)	1638 (0 to 3879)

Sample sizes ranged from 2336 to 2415.

The dimensionality of the scales measuring ‘perceptions of the local area’ [28], and the ‘presence of recreational facilities within the local area’ [29] was examined using exploratory factor analysis with a varimax (orthogonal) rotation. Factor analysis examines whether the variation in the observed variables can be explained largely by a smaller number of underlying factors. For the scale measuring perceived environmental characteristics the scree plot indicated there were three factors “Traffic and pleasantness of surroundings”, “Proximity and convenience of walking”, and “Safety and convenience of cycling”. Two original scale items (“Convenience of public transport” and “Safety of walking after dark”) were not strongly correlated with any of the factors (factor loadings <0.5) and so were treated as separate variables. Two factors were indicated for the scale measuring availability of recreational facilities in the local area. These were “Manmade sports facilities in local area” and “Natural activity facilities in local area”. The item “Community centre/village hall in local area” was treated as a separate variable, because the factor loading was less than 0.5. Composite scores were created for each of the factors, based on the mean of the items that had their primary loadings on each factor.

### Meets recommended activity guidelines

The logistic regression analyses (Table 3) revealed that being male and in better health were positively associated with the odds of meeting the recommended activity guideline in the fully adjusted models. Greater commitment to doing more physical activity, favourable activity social norms, and a greater physical activity habit were associated with increased odds of being active at recommended levels in the fully adjusted model. Recent use of recreational facilities was also associated with meeting the guidelines.

**Table 3:** Odds ratios for meeting physical activity guidelines – logistic regression

			***Unadjusted***			***Fully adjusted***	
***Predictor variable***		***OR***	***95*%** ***CI***	***p***	***OR***	***95*%** ***CI***	***p***
**Personal factors**							
	Gender			0.03			0.002
	*Male*		Reference			Reference	
	*Female*	0.82	0.69 to 0.98		0.70	0.55 to 0.88	
	Age groups (years)			<0.001			0.54
	*18-34*		Reference			Reference	
	*35-49*	0.87	0.57 to 1.33		0.99	0.61 to 1.61	
	*50-64*	0.70	0.47 to 1.04		0.99	0.62 to 1.58	
	*65+*	0.32	0.21 to 0.48		0.82	0.50 to 1.35	
	BMI category			<0.001			0.40
	*Normal weight*		Reference			Reference	
	*Overweight*	0.79	0.65 to 0.96		0.93	0.74 to 1.18	
	*Obese*	0.47	0.37 to 0.60		0.81	0.59 to 1.10	
	Health			<0.001			<0.001
	*Poor/fair*		Reference			Reference	
	*Good*	2.83	2.23 to 3.61		1.57	1.14 to 2.17	
	*Very good/excellent*	5.92	4.66 to 7.53		2.05	1.44 to 2.91	
	Long-term illness/disability			<0.001			0.06
	*Yes*		Reference			Reference	
	*No*	3.27	2.71 to 3.94		1.31	0.99 to 1.73	
	Education leaving age (years)			<0.001			0.98
	*16 & under*		Reference			Reference	
	*17-18*	1.58	1.27 to 1.96		1.02	0.78 to 1.34	
	*19+*	1.86	1.52 to 2.28		1.00	0.77 to 1.28	
	Occupation category			<0.001			
	*Not employed*		Reference				
	*Sitting/standing job*	1.82	1.51 to 2.20				
	*Physical job*	2.41	1.82 to 3.20				
	Cars in household			<0.001			0.22
	*No car*		Reference			Reference	
	*1 car*	4.11	2.55 to 6.62		1.38	0.71 to 2.66	
	*2+ cars*	7.74	4.82 to 12.43		1.61	0.82 to 3.17	
	Children under 15 in household			<0.001			
	*Yes*		Reference				
	*No*	0.58	0.47 to 0.73				
	Dog ownership			<0.001			0.15
	*Yes*		Reference			Reference	
	*No*	0.71	0.60 to 0.85		0.85	0.68 to 1.06	
**Social factors**							
	Commitment to doing more physical activity			<0.001			0.002
	*Low*		Reference			Reference	
	*Moderate*	1.63	1.33 to 1.99		1.21	0.94 to 1.55	
	*High*	2.79	2.22 to 3.50		1.66	1.25 to 2.20	
	Physical activity social norms			<0.001			0.004
	*Unfavourable*		Reference			Reference	
	*Neutral*	1.43	1.13 to 1.80		1.05	0.79 to 1.40	
	*Favourable*	2.53	2.06 to 3.11		1.47	1.14 to 1.90	
	Physical activity habit			<0.001			<0.001
	*Unfavourable*		Reference			Reference	
	*Neutral*	2.17	1.57 to 3.02		1.61	1.12 to 2.33	
	*Favourable*	7.77	6.27 to 9.62		4.30	3.33 to 5.55	
	Physical activity village supportiveness			<0.001			
	*Unfavourable*		Reference				
	*Neutral*	1.66	1.33 to 2.07				
	*Favourable*	1.95	1.57 to 2.41				
**Environmental factors**							
	Traffic and pleasantness of surroundings			0.04			
	*Unfavourable*		Reference				
	*Neutral*	1.15	0.83 to 1.62				
	*Favourable*	1.37	1.06 to 1.76				
	Proximity and convenience of walking			0.30			
	*Unfavourable*		Reference				
	*Neutral*	1.17	0.90 to 1.53				
	*Favourable*	0.96	0.80 to 1.16				
	Safety and convenience of cycling			0.96			
	*Unfavourable*		Reference				
	*Neutral*	1.02	0.83 to 1.25				
	*Favourable*	0.98	0.79 to 1.22				
	Convenience of public transport			0.18			0.64
	*Unfavourable*		Reference			Reference	
	*Neutral*	0.94	0.73 to 1.21		0.93	0.68 to 1.27	
	*Favourable*	0.83	0.68 to 1.01		0.89	0.70 to 1.14	
	Safety walking after dark			0.002			
	*Unfavourable*		Reference				
	*Neutral*	0.97	0.75 to 1.27				
	*Favourable*	1.36	1.11 to 1.66				
	Manmade sports facilities in local area			<0.001			
	*1+ facility*		Reference				
	*No facilities*	0.72	0.60 to 0.85				
	Natural activity facilities in local area			<0.001			
	*1+ facility*		Reference				
	*No facilities*	0.35	0.22 to 0.58				
	Community centre in local area			<0.001			
	*Yes*		Reference				
	*No*	0.66	0.54 to 0.82				
	Use of recreational facilities			<0.001			0.01
	*No facilities used*		Reference			Reference	
	*Used in last year only*	1.70	1.12 to 2.58		1.31	0.70 to 2.44	
	*Used in last month*	4.49	3.19 to 6.33		2.04	1.10 to 3.47	
	Locality of facilities used			<0.001			0.27
	*No facilities used*		Reference			Reference	
	*Local village only*	1.32	1.01 to 1.72		0.73	0.48 to 1.11	
	*Outside local village*	2.28	1.66 to 3.12		0.97	0.61 to 1.54	
	*Local and not local*	2.31	1.81 to 2.94		0.88	0.59 to 1.31	
**Village-level factors**							
	Population density	1.07	0.92 to 1.27	0.36			
	Mean age	1.01	0.99 to 1.04	0.27			
	Gender (% males)			0.18			
	*Low*		Reference				
	*Moderate*	1.14	0.93 to 1.41				
	*High*	0.94	0.77 to 1.15				
	IMD score	0.99	0.97 to 1.01	0.47			
	Sport England Segmentation			0.48			
	*S19 (Elsie & Arnold)*		Reference				
	*S17 (Ralph & Phyllis)*	0.84	0.52 to 1.36				
	*S13 (Roger & Joy)*	0.69	0.41 to 1.18				
	*S11 (Philip)*	0.49	0.23 to 1.05				
	*S8 (Jackie)*	0.91	0.19 to 4.25				
	*S6 (Tim)*	0.72	0.43 to 1.22				
	*S3 (Chloe)*	0.65	0.36 to 1.18				

Sample sizes for the unadjusted analyses ranged from 2336 to 2415; sample size for the adjusted analysis was 2174.

‘Commitment to doing more physical activity’ was the only variable found to have a significant interaction with gender (p-value for interaction = 0.04). There was little evidence of an association between commitment to doing more physical activity and meeting the recommended activity guideline for females (p = 0.19). Males, however, with ‘moderate’ (adjusted OR 1.52, 95% CI: 1.03 to 2.24) or ‘high’ (adjusted OR 2.64, 95% CI: 1.59 to 4.38) commitment levels, had increased odds of meeting the guidelines, compared to those with ‘low’ commitment levels (p < 0.001).

### Total leisure-time physical activity

The linear regression analyses revealed that being male, under 35, of normal body mass index, and in good health, were all associated with increased leisure-time physical activity (LTPA; Table 4). In terms of occupational activity, people with sitting or standing occupations did *less* MET-minutes per week of physical activity than people who were not employed. People with physical jobs did the most LTPA per week. Owning a dog was also associated with increased LTPA. Participants with moderate ‘commitment to doing more physical activity’ levels reported the least LTPA. Positive activity social norms and physical activity habits were associated with increased leisure-time physical activity. Inconvenience of public transport and using facilities outside the local village were both associated with increased leisure-time physical activity behaviour in the fully adjusted model.

**Table 4:** Regression coefficients for MET-minutes/week physical activity (total LTPA) – linear regression

			***Unadjusted***			***Fully adjusted***	
***Predictor variable***		***Coeff.***	***95*%** ***CI***	***p***	***Coeff.***	***95*%** ***CI***	***p***
**Personal factors**							
	Gender			<0.001			<0.001
	*Male*		Reference			Reference	
	*Female*	-519	-763 to -274		-597	-841 to -352	
	Age groups (years)			<0.001			0.002
	*18-34*		Reference			Reference	
	*35-49*	-864	-1378 to -351		-694	-1182 to -206	
	*50-64*	-596	-1088 to -103		-368	-843 to 108	
	*65+*	-1249	-1744 to -754		-787	-1318 to -255	
	BMI Category			<0.001			0.02
	*Normal weight*		Reference			Reference	
	*Overweight*	-540	-804 to -277		-365	-618 to -111	
	*Obese*	-879	-1232 to -525		-195	-540 to 151	
	Health			<0.001			<0.001
	*Poor/Fair*		Reference			Reference	
	*Good*	939	601 to 1278		505	153 to 857	
	*Excellent/Very good*	1765	1444 to 2086		836	481 to 1190	
	Long-term Illness/Disability			<0.001			
	*Yes*		Reference				
	*No*		687 to 1208				
	Education leaving age (years)			0.004			
	*16 & under*		Reference				
	*17-18*	449	146 to 752				
	*19+*	379	103 to 654				
	Occupation category			<0.001			<0.001
	*Not employed*		Reference			Reference	
	*Sitting/standing job*	-84	-341 to 173		-526	-831 to -222	
	*Physical job*	1274	921 to 1627		530	147 to 912	
	Cars in household			<0.001			
	*No car*		Reference				
	*1 car*	1143	516 to 1770				
	*2+ cars*	1627	1009 to 2244				
	Children under 15 in household			0.98			
	*Yes*		Reference				
	*No*	-4	-292 to 285				
	Dog ownership			<0.001			0.03
	*Yes*		Reference			Reference	
	*No*	-527	-770 to -284		-262	-501 to -23	
**Social factors**							
	Commitment to doing more physical activity			0.002			0.03
	*Low*		Reference			Reference	
	*Moderate*	-114	-398 to 170		-317	-599 to -36	
	*High*	403	111 to 696		18	-286 to 322	
	Physical activity social norms			<0.001			<0.001
	*Unfavourable*		Reference			Reference	
	*Neutral*	474	140 to 807		154	-170 to 478	
	*Favourable*	1096	813 to 1379		513	228 to 799	
	Physical activity habit			<0.001			<0.001
	*Unfavourable*		Reference			Reference	
	*Neutral*	814	361 to 1267		447	-16 to 910	
	*Favourable*	2245	1974 to 2516		1557	1244 to 1870	
	Physical activity village supportiveness			<0.001			
	*Unfavourable*		Reference				
	*Neutral*	409	109 to 708				
	*Favourable*	553	273 to 833				
**Environmental factors**							
	Traffic and pleasantness of surroundings			0.06			
	*Unfavourable*		Reference				
	*Neutral*	-139	-618 to 339				
	*Favourable*	257	-110 to 624				
	Proximity and convenience of walking			0.02			
	*Unfavourable*		Reference				
	*Neutral*	397	37 to 757				
	*Favourable*	-83	-351 to 185				
	Safety and convenience of cycling			0.14			
	*Unfavourable*		Reference				
	*Neutral*	109	-176 to 395				
	*Favourable*	304	-1 to 608				
	Convenience of public transport			0.06			0.04
	*Unfavourable*		Reference			Reference	
	*Neutral*	-25	-382 to 332		-52	-391 to 287	
	*Favourable*	-336	-619 to -52		-348	-617 to -80	
	Safety walking after dark			0.003			0.22
	*Unfavourable*		Reference			Reference	
	*Neutral*	-224	-601 to 152		-90	-451 to 271	
	*Favourable*	304	24 to 585		164	-108 to 436	
	Manmade sports facilities in local area			0.06			
	*1+ facility*		Reference				
	*No facilities*	-240	-493 to 14				
	Natural activity facilities in local area			0.02			
	*1+ facility*		Reference				
	*No facilities*	-864	-1592 to -135				
	Community centre in local area			0.003			0.11
	*Yes*		Reference			Reference	
	*No*	-450	-749 to -152		-239	-527 to 50	
	Use of recreational facilities			<0.001			0.05
	*No facilities used*		Reference			Reference	
	*Used in last year only*	69	-530 to 668		-126	-833 to 580	
	*Used in last month*	1083	605 to 1561		351	-294 to 997	
	Locality of facilities used			0.001			0.007
	*No facilities used*		Reference			Reference	
	*Local village only*	43	-344 to 429		-263	-714 to 189	
	*Outside local village*	775	330 to 1220		297	-198 to 791	
	*Local and not local*	318	-30 to 666		-286	-709 to 137	
**Village-level factors**							
	Population density	20	-242 to 281	0.88			
	Mean age	14	-28 to 56	0.52			
	Gender (% males)			0.24			
	*Low*		Reference				
	*Moderate*	294	-49 to 636				
	*High*	125	-218 to 469				
	IMD score	3	-32 to 39	0.85			
	Sport England Segmentation			0.38			
	*S19 (Elsie & Arnold)*		Reference				
	*S17 (Ralph & Phyllis)*	-46	-779 to 688				
	*S13 (Roger & Joy)*	-127	-948 to 695				
	*S11 (Philip)*	-567	-1810 to 676				
	*S8 (Jackie)*	1091	-1187 to 3369				
	*S6 (Tim)*		-260	-1064 to 544			
	*S3 (Chloe)*	-652	-1592 to 288				

Sample sizes for the unadjusted analyses ranged from 2336 to 2415; sample size for the adjusted analysis was 2179.

‘Convenience of public transport’ was the only variable that had a significant interaction with gender (p-value for interaction = 0.04). There was little evidence of an association between convenience of public transport and total leisure-time physical activity for females (p = 0.14). Males, however, with ‘neutral’ (adjusted mean difference = -508, 95% CI: -1061 to 45) or ‘favourable’ (adjusted mean difference = -524, 95% CI: -959 to -90) opinions about the convenience of public transport did less leisure-time physical activity than those with ‘unfavourable’ opinions on the convenience of public transport (p = 0.03).

### Village-level factors

None of the village-level factors were significantly associated with reported leisure-time physical activity.

### Ancillary analysis

An ancillary analysis was conducted for the ‘commitment to do more physical activity’ variable. It was hypothesised that the lack of association between ‘commitment to do more physical activity’ and total reported leisure-time physical activity was due to the majority of participants being sufficiently physically active, and therefore having low commitment levels to do more physical activity. To investigate this, the unadjusted and fully adjusted regression models were repeated with only those participants who did not report doing sufficient activity to meet the recommended guidelines (Table 5). Commitment to doing more physical activity was significantly positively associated with LTPA in the unadjusted model, but not in the fully adjusted model.

**Table 5:** Regression coefficients for MET-minutes/week physical activity (participants who didn’t meet the recommended guidelines) – linear regression

		***Unadjusted***			***Fully adjusted***	
***Predictor variable***	***Coeff.***	***95*%** ***CI***	***p***	***Coeff.***	***95*%** ***CI***	***p***
Commitment to doing more physical activity			<0.001			0.19
Low		Reference			Reference	
Moderate	47	15 to 80		10	-26 to 46	
High	99	60 to 137		40	-4 to 85	

### Village- and participant-level variation

Only 2.4% of the variation in reported leisure-time physical activity was at the village level (i.e., 97.6% was at the participant level). The fully adjusted model explained 72.6% of the between-village variation and 18.7% of the participant-level variation in physical activity.

## Discussion

The purpose of this study was to examine the personal, social, and environmental correlates of physical activity in rural adults from the United Kingdom. A number of variables were identified as correlates of physical activity behaviour. Gender, health status, commitment to doing more physical activity, social norms, physical activity habit, and reported use of recreational facilities were all associated with both meeting the recommended guidelines and total reported LTPA. Age, BMI, occupational activity, dog ownership, locality of recreational facilities, and convenience of public transport were only correlates for total LTPA.

Although cross-sectional data are useful for identifying associations, analyses of longitudinal data provide a stronger basis for inferring causality [37,38]. In one review, Bauman et al. [37] identified health status as one of the clearest predictors of change in physical activity behaviour in adults. There was also consistent evidence to suggest personal history of physical activity during adulthood [38,39] (similar to ‘physical activity habit’), and intention to exercise [38-40] (similar to ‘commitment to do more physical activity’), were both predictors of change in physical activity behaviour. Reviews suggest that social norms are not associated with physical activity behaviour [37]. Therefore, findings from the present study imply that rural populations are similar to the general population in terms of the association between health status, physical activity habit, commitment to be more active, and their reported physical activity behaviour. The association between social norms and physical activity in the present study suggests, however, that social norms may be a uniquely important factor for rural populations.

Other correlates of physical activity reported in the literature are male sex [39,40], age (negatively) [39-41], and overweight (negatively) [39]. Our findings concur with this research, although age and overweight status were only associated with total leisure-time physical activity and not the likelihood of meeting the guidelines. In line with previous research, dog owners report more physical activity than people who do not own dogs [42-44].

Accessibility of recreational facilities has been found to be the most consistent environmental predictor of physical activity and change in physical activity behaviour in reviews [37,38,45,46]. In the present study, how recently participants had used recreational facilities, and the locality of facilities used, were both associated with physical activity behaviour. Logically, the more recently participants had used a recreational facility, the more likely they were to have met the recommended guidelines. Research from urban populations has found that local recreational facilities are visited more frequently than those located further away [47,48]. In our study, the mixed outcome for locality of facilities used suggests that it is less important for rural populations where facilities are located. It may be suggested that rural adults have to travel to use facilities because there are limited facilities available within local villages. However, in fact, nearly all participants (97%) perceived there to be at least one natural activity facility in their local area, with 61% perceiving there to be at least one man-made sports facility. It, therefore, seems that recreational facilities were available in these rural locations. Although some facilities may have been available locally, this does not necessarily mean residents used them regularly. It is plausible that if individuals had a desire to do a particular activity that was not offered locally, or had a personal preference for a certain facility, they might have been willing to travel the necessary distance. This finding warrants further investigation, in order to understand whether rural adults would benefit from more recreational facilities in their local village.

Convenience of public transport was negatively associated with leisure-time physical activity. This finding contradicts a recent review paper that found greater access to public transport to be positively associated with walking behaviour [49]. This may be due in part to the limited public transport services available in rural Devon, with 59% of participants reporting unfavourable responses for the convenience of public transport. Additionally, this study only measured convenience of public transport, rather than use. Thus, it may be that individuals who regularly used public transport also did more walking than individuals who did not.

### Strengths and limitations

Two key strengths of this study are the large sample size (n = 2,415), and the random selection of participants. Additionally, the study examined a range of personal, social and perceived environmental factors, in addition to village-level factors. Although this study forms part of a longitudinal study, the data presented here are cross-sectional and, therefore, can only be used to examine associations rather than to draw inferences regarding causality. Despite being better than anticipated, and comparing well with other survey studies from the United Kingdom (15.9% [28], 17% [35]), the response rate was low (37.7%). This raises concerns that those who consented may not represent the wider population (non-response bias) [50]. However, the participants in the present study were similar to the wider population in terms of IMD score and the population density of the village they resided in. Compared to the wider population, however, the survey respondents tended to be older, with a greater proportion being female. Previous research suggests females and older adults are often over-represented in health surveys [4]. Two-thirds of the population reported meeting the recommended guidelines, suggesting that those of higher activity levels tend to be over-represented. Whilst an unrepresentative sample is compromised when estimating a mean or prevalence, such data are generally robust for examining relationships between variables, in this case between physical activity and potential correlates. A further limitation of this study is the use of self-reported data. We used established and validated measures where possible, but although the IPAQ-SV has been found to have acceptable levels of test-retest reliability (r = 0.76) [23], recent reviews have questioned its levels of criterion validity (ρ = 0.30, 95% CI 0.23 to 0.36 [22]; median ρ = 0.29, range 0.09 to 0.39 [24]). Self-report measures of physical activity tend to include bias due to social desirability and participants may find it difficult to recall activities from the past seven days. The fact that self-reported height and weight were used to calculate body mass index is another limitation, because of social desirability bias to over-report height and under-report weight [51]. Despite this, Goodman and Strauss [52] stated that self-report measures are acceptable in epidemiological studies given that self-report measures are correlated with measured height and weight. Finally, participants were not asked about their ethnic origin in the questionnaire. This was, however, a deliberate decision, because only 2.5% of the rural population of Devon is from non-white British ethnic groups [53].

### Implications

Despite the noted limitations, our findings are important from a public health perspective, in terms of understanding the unique characteristics of rural populations, through focusing on the personal, social, and environmental correlates of physical activity. Regular physical activity plays a key role in reducing the risk factors for several chronic conditions. Therefore, the identification of physical activity correlates may help researchers, clinicians, and health policy makers to design population-specific interventions. This study adds to the limited research available on physical activity in rural communities from England. The results from the present study suggest that rural populations are similar to urban populations in terms of the correlates of physical activity behaviour. However, our findings do imply that social norms may be more influential for rural populations, compared to their urban counterparts. Contradictory to research from urban populations, there was a negative association between convenience of public transport and physical activity, and the most active individuals used recreational facilities exclusively outside of their local area. These findings suggest that rural and urban adults differ in terms of the way they interact with their environment, and that differences in the built environment have an influence on physical activity behaviour. To successfully change physical activity prevalence in rural populations, interventions should be tailored to modify the correlates of physical activity behaviour that are specific to rural adults, as identified in the present study.

### Future research

Future research should focus on longitudinal studies with rural populations to examine the determinants of physical activity behaviour, to aid the understanding of the causal role and direction of effect of correlates. It is also recommended that the physical activity correlates from this and other similar studies be used to help develop future physical activity interventions specifically tailored to rural communities, and that rigorous evaluation methods be undertaken to determine the effectiveness of such programmes.

## Conclusions

This study aimed to examine the personal, social and environmental correlates of physical activity behaviour in rural adults from south-west England. Both individual and village-level predictors were included in the analysis, with gender, health, commitment to being more active, activity habits, social norms, and use of recreational facilities revealed as the clearest correlates of physical activity behaviour. Although most of the results were in line with previous research, this study did highlight some unique characteristics of the rural population. Understanding the correlates that influence physical activity behaviour is important for the designing of effective physical activity interventions, but generally the relationship between these correlates is complex and typically understudied, especially in rural populations.

## Abbreviations

BMI: Body mass index; IPAQ-SV: International physical activity questionnaire – short version; LTPA: Leisure-time physical activity; MET: Metabolic equivalent.

## Competing interests

The authors declare that they have no competing interests.

## Authors’ contributions

The study’s chief investigators ES, MH and TR were responsible for identifying the research question, the design of the study, obtaining ethics approval and the acquisition of funding. OCU contributed to the fine-tuning of the methodology and conducted the randomisation procedures. ES carried out the data collection and processing. OCU, BM, MH and ES contributed to the statistical analysis. All authors helped draft and revise the manuscript and approved the final version.
